# Preparation of β-CD-DPPE-Dox Nanomedicine and Its’ Application as the Anticancer and Antitumor Drug

**DOI:** 10.1038/s41598-019-50162-8

**Published:** 2019-09-20

**Authors:** Miaomiao Yan, Anran Cai, Jing Li, Meixiu Xin, Mingying Liu, Chunhua Wang, Guangcheng Wei

**Affiliations:** Department of Pharmacy Science, Binzhou Medical University, Yantai, 264003 China

**Keywords:** Molecular medicine, Drug delivery

## Abstract

β-CD-DPPE molecule was synthesized through the conjugation of β-CD-NH_2_ and the DPPE molecule, and its’ water-solubility was more excellent than the traditional phospholipid molecule. The spherical micelles was formed by β-CD-DPPE molecule in aqueous solution, and the β-CD-DPPE-Dox nanomedicine can be prepared through loading Dox (Doxorubicin) into the micelles, and the Dox loading ratio was about 82.3 ± 7.27%. At the same time the Dox release behavior from the nanomedicine was sustained-release and pH controlled release, and the release test *in vitro* showed that the release rate of the Dox at the lower pH was faster than that of normal pH (pH = 7.4), which indicated that the rate of release in the tumor microenvironment is faster than in the normal tissue. Biological test showed that the micelles was low cytotoxicity, and the cytotoxicity of β-CD-DPPE-Dox nanomedicine was lower than the Dox under the same Dox concentration, and the β-CD-DPPE-Dox nanomedicine could effectively induce cancer cell apoptosis and inhibit the tumor growth.

## Introduction

At present, clinical treatments for cancer include chemotherapy, surgery, radiotherapy, and immunotherapy^1–6^. Chemotherapy is a common method for cancer treatment^7^, however, chemotherapy drugs used in chemotherapy limit their clinical applications because of their inherent properties, such as non-specific activity and non-specific bio-distribution, and the chemotherapeutic drugs can kill the cancer cells but also damage the normal tissues, especially heart^8^, bone marrow^9^, gastrointestinal tract^10^, and nervous system^11^. To solve this problem, a series of drug delivery systems have been extensively studied, such as micelles, liposomes, dendrimers, and gels^12^. Liposomes are closed vesicles with a bilayer structure, which can loaded with hydrophilic drugs or hydrophobic drugs also, can increase the water solubility and stability of the loaded drugs, and can reduce the toxicity of pure anticancer drugs when they circulate *in vivo*. In 1995, liposomes PEG-modified were marketed in the United States. However, with the application of PEG modified liposomes, there was appeared adverse reaction called “hand foot syndrome”^13^. At the same time, it has been found that PEG-modified liposomes reduce the interaction between liposomes and cell membranes, impede the ability of the liposomes to enter the tumor tissue, and results in the accumulation decreasing of liposomes in the tumor tissue. In order to get a safer, more stable, and more efficient liposomes, we designed and synthesized a novel phospholipid molecules modified by β-cyclodextrin which could self-assembled into micelles, and furthermore it was applied as the drug delivery system. Cyclodextrin which have good biological properties and unique structure have received extensive attention in the field of drug delivery system and prodrug^14^. Beta-cyclodextrin (β-CD) is a cyclic oligosaccharide composed of seven glucopyranose units, and it has excellent biocompatibility. β-CD has hydrophilic shell and hydrophobic cavity which is ideal harbors for fat-soluble drugs. β-CD can increase the solubility and safety of fat-soluble drugs while increasing the bioavailability of loaded drugs^15^.

**Figure 1:**
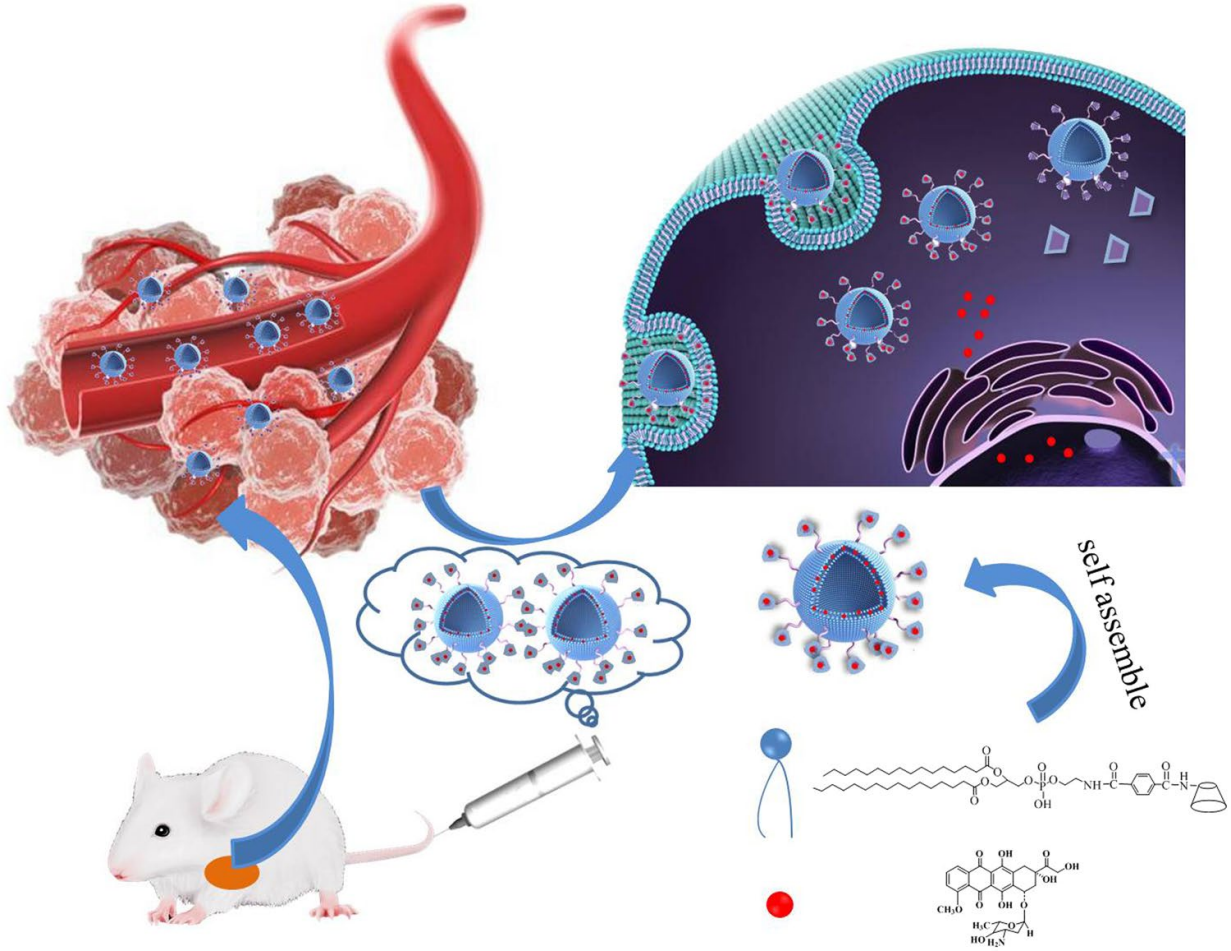
β-CD-DPPE molecules self-assemble into micelles which is used as drug delivery system to form nanomedicine for the treatment of tumor.

In this system (Fig. 1), phospholipid molecules (DPPE) and β-CD molecules (β-CD-NH_2_) were covalently conjugated to form a novel amphiphilic β-CD-DPPE molecule which significantly increased the water solubility of phospholipids, and β-CD-DPPE molecule could self-assemble into β-CD-DPPE micelles in aqueous solution. The release of Dox from the β-CD-DPPE micelles had the characteristics of pH response and sustained release. Cell experiments have shown that the β-CD-DPPE liposome had very good biocompatibility and extremely low biological toxicity. The β-CD-DPPE-Dox nanomedicine could effectively induce cancer cell apoptosis. At the same time, animal experiments showed that the β-CD-DPPE-Dox nanomedicine could effectively inhibit the growth of the tumor and even significantly reduce the tumor volume without affecting the body weight of the mouse. The tumor tissue section shows pyknosis and apoptosis of the cancer cell, and large areas of tumor tissue also necrosis, and it further proved that β-CD-DPPE-Dox nanomedicine could effectively inhibit tumor growth, and achieve the treatment purpose of tumors.

## Experimental

### Preparation of terephthalic acid methyl ONSu ester

**Figure 2:**
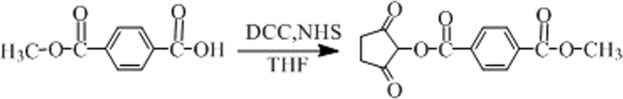
The synthesis illustration of terephthalic acid methyl ONSu ester.

*N-*hydroxylsuccinimide (NHS, 2.4 g, 18.0 mmol), dicyclohexyl carbodiimide (DCC, 3.7 g, 18.0 mmol), and terephthalic acid monomethyl ester (2.2 g, 12.0 mmol) were added to THF solution (30 mL) in round bottom flask to react at room temperature. After 48 h, the precipitate, dicyclohexylurea (DCU), was removed by centrifuge. The white solid powder was obtained through the vacuum freeze-drying to the supernatant solution^16^ (Fig. 2).

### Synthesis of methylterephthalamide β-CD

**Figure 3:**
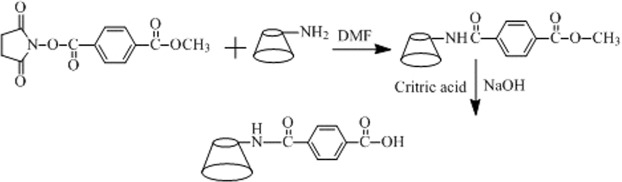
The synthetic route of β-CD-NH-CO-C_6_H_4_-COOH.

^**1**^**HNMR** (DMSO-d_6_, 25, 500 MHz): δ8.25 (t, 1H,-CONH-), δ8.1(s, 1H,-COOH), δ8.0 (d, 2H, 2-*Ph*), δ7.95(d, 2H, 3-*Ph*), δ4.9–4.3(m, 6H, (C(1)H)), δ4.6–4.8(m, 3H, O(6)H), δ3.5–4.1(m, 14H, C(2)H and C(4)H). **MALDI-TOF MS (*m*/*z*)**: 1305.16 ([M + Na]^+^).

6A-Amino-3A-deoxy-(2AS,3AS)-beta-cyclodextrin Hydrate (NH_2_-β-CD, 1.0 g, 0.88 mmol) and Terephthalic acid methyl ONSu ester (0.5 g, 1.80 mmol) was added to 20 mL DMF with stirring for 36 h^16^ until the ivory-white solution became clear solution, and then the white solid powder (β-CD-NH-CO-C_6_H_4_-COOCH_3_) was obtained through the vacuum freeze-drying to the clear solution. The crude sample (β-CD-NH-CO-C_6_H_4_-COOCH_3_) was firstly dissolved in 10 mL water, and then 0.2 mL of 1 mol·L^−1^ NaOH was added, and the sample was hydrolyzed with stirring for an hour. The reaction mixture was neutralized with citric acid, and then crude product (β-CD-NH-CO-C_6_H_4_-COOH) was obtained through the vacuum freeze-drying to the mixture solution. The crude product was purified by column chromatography with DIAION HP-20 (The ratio of eluent with water/methanol from 100/0 to 50/50). The 60/40 (water/methanol) eluent was concentrated to give the pure sample β-CD-NH-CO-C_6_H_4_-COOH in 85% yield. NMR (Fig. S1), MS (Fig. S2) and FT-IR (Fig. S3) were used to determined the molecular structure (Fig. 3).

### Synthesis of β-CD-NH-CO-C_6_H_4_-CONH-DPPE (β-CD-DPPE) compound

**Figure 4:**
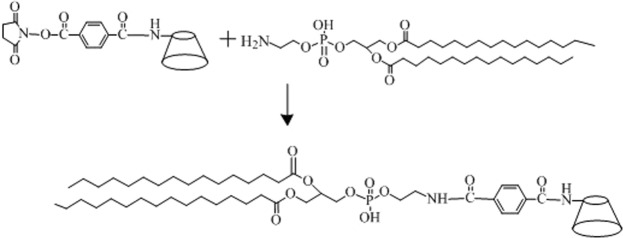
The synthetic route of β-CD-DPPE molecule.

^**1**^**H NMR** (500 MHz, CDCl_3_) δ 8.35 (s, 1 H,-CO-NH-), δ 7.49(CD-NH-CO-), δ 7.28 (m, 2H, 2,3-*Ph*), δ5.71–4.83 (m, 1 H), δ5.25 (m, 1H,-CH-CO-), δ4.39 (m, 3 H,-O-CH_2_-CH-), δ4.0–4.2 (m, 2 H,-CH_2_-O-), δ4.19 (s, 2 H), δ4.19–3.90 (m, 4 H,-CH_2_-CH_2_-), δ3.25 (s, 1 H,-OH). **MALDI-TOF MS (*m*/*z*):** 1957.11 ([M + H]^+^).

β-CD-NH-CO-C_6_H_4_-COOH (0.46 g, 6.60 mmol), DCC (0.185 g, 0.90 mmol) and NHS (0.103 g, 0.9 mmol) was added to 20 mL anhydrous DMF with stirring for 48 h at room temperature, the precipitate was removed by centrifuge. Then the L-DPPE (0.43 g, 0.62 mmol) which was dissolved in 20 mL THF was mixed with the above supernatant solution with stirring for 24 h at room temperature. After the prescribed time, the supernatant solution was collected, and then white solid powder was obtained through the vacuum freeze-drying. The crude product was purified by column chromatography with silica gel column (The ratio of eluent with dichloromethane to methanol from 75/25 to 30/70). The pure sample β-CD-DPPE was obtained through the sample characterization analysis in 83% yield. NMR (Fig. S4), MS (Fig. S5) and were used to determine the molecular structure (Fig. 4).

### The measurement of critical micelle concentration of amphiphilic β-CD-DPPE molecules

The β-CD-DPPE molecule could self-assemble into β-CD-DPPE micelles in aqueous solution. The CMC of the micelles was measured by pyrene fluorescence probe which was a kind of hydrophobic fluorescence probe^17^. Briefly, the pyrene was put into volumetric flask with the concentration 6 × 10^−7^ mol·L^−1^, at the same time a series of β-CD-DPPE different concentration solution from 1 × 10^−4^ mg·mL^−1^ to 1 × 10^−1^ mg·mL^−1^ was added separately. The mixed solution was placed into the water bath for 30 min, and the fluorescent intensity of the solution was recorded by fluorescence spectrophotometer (LS-55, PerkinElmer, U.K.) at 295 nm (Excitation slit 5.0 nm and emission slit 2.5 nm). The CMC value was evaluated by the pyrene fluorescence intersection at 371 nm (I_371_) and 391 nm (I_391_) with a rapidly reduced ratio value of I_371_/I_391_ as shown in Fig. S6.

### Preparation of β-CD-DPPE-Dox nanomedicine

Doxorubicin (Dox, 1 mg) was dissolved into 10 mL methanol solution which contained triethylamine (The molar ratio of Dox to triethylamine is 1 to 2). β-CD-DPPE was dissolved in organic solvent (The volume ratio of dichloromethane to methanol is 1 to 1). Then the above two solution was mixed in eggplant shaped bottle and incubated for 30 min at 50 °C. After the prescribed time, the phospholipid membrane was formed around the inner surface of eggplant shaped bottle after removing the organic solvent, and then the PBS (1X, pH = 7.4) was added to the eggplant shaped bottle, and ultrasonic, then the precipitate was collected by centrifuge. The supernatant solution was used to calculate the encapsulation efficiency (EE%) and drug loading (DL%) through determining the absorbance intensity at 485 nm according to the following formulas.

$$EE \% =(1-\frac{{C}_{sup}}{{{\rm{C}}}_{{\rm{tol}}}})\times 100 \% $$

$$DL \% =\frac{{\rm{weight}}\,{\rm{of}}\,{\rm{Dox}}\,{\rm{encapsulated}}\,{\rm{in}}\,{\rm{micelles}}}{{\rm{total}}\,{\rm{weight}}\,{\rm{of}}\,{\rm{micelles}}}\times 100 \% $$

### Morphology characterization and size distribution of micelles

Micelles morphology was observed with the transmission electron microscope (TEM, JEM-1400), and the micelles morphology was firstly stained with the phosphotungstic acid for 2 minutes before observation. The micelles size distribution and Zeta potential were determined with Malvern laser particle size analyzer (Malver, UK).

The stability of β-CD-DPPE aggregates in normal saline (Contain 10% V/V serum) was also determined with the Malvern laser particle size analyzer through determining the size change of β-CD-DPPE aggregates in normal saline (NS) solution with the time increasing.

### Dox release behavior from β-CD-DPPE-Dox nanomedicine *in vitro*

Tumor tissue has obvious pH gradient compared with normal tissue^18^, and generally speaking, pH value of tumor tissue is lower than normal tissue. Dox as the model drug was chosen to study the release behavior from the micelles. To simulate the normal body fluid environment and lower pH tumor environment, we studied the release behavior of Dox under pH = 5.0 and pH = 7.4, and in addition, the effects of temperature on release behavior at 25 °C and 37 °C were also studied. A certain amount of micelles which loaded Dox was dissolved in 4 mL 1X PBS, and then added into dialysis bag (MW 8000D) to dialysis in 20 mL PBS. The absorbance intensity was determined at 485 nm with the time increasing, and then the solution was re-poured into the dialysis bag after determination.

### Cell culture

MCF-7 and HepG2 cell lines were the gift which came from the medical research center of Binzhou Medical University. MCF-7 and HepG2 were cultured in DMEM high glucose medium which contained10% fetal bovine serum and 1% antibiotics (Streptomycin, 100 U·mL^−1^, Penicillin). These two cell lines were incubated in 5% carbon dioxide incubator at 37 °C.

#### Analysis of cytotoxicity and anticancer activity *in vitro*

Cytotoxicity evaluation of β-CD-DPPE micelles and anticancer activity of β-CD-DPPE-Dox nanomedicine *in vitro* were performed by MTT assay^19^. HepG2 and MCF-7 cell lines were chosen for the analysis of cytotoxicity and anti-cancer activity *in vitro*, and the reason is that the Dox is a broad-spectrum antitumor drug, and it is effective for many cancers lines, including MCF7 and HepG2 cells lines which are widely used to evaluate the effect as many literatures reported. In this experiment, untreated cells were used as a control group and cell viability was calculated using the following formula:

$$\begin{array}{rcl}{\rm{Cell}}\,{\rm{survival}}\,{\rm{rate}}\,( \% ) & = & {\rm{Absorbance}}\,{\rm{intensity}}\,{\rm{of}}\,{\rm{the}}\,{\rm{experimental}}\,{\rm{group}}\\  &  & ({{\rm{OD}}}_{{\rm{treated}}})/{\rm{Control}}\,{\rm{group}}\,{(\mathrm{OD}}_{{\rm{control}}})\times 100.\end{array}$$

Each sample was determined for three times, and the significant level was set as P < 0.05.

#### Cell uptake experiment

The quantitative analysis of cell uptake to β-CD-DPPE-Dox nanomedicine was performed with flow cytometry (FC)^19^, and HepG2 cells was used to execute the quantitative analysis. The assay was performed with a FC (EPICS XL, Beckman, USA), and the excitation wavelength is 488 nm and the emission wavelength is 590 nm. The distribution of β-CD-DPPE-Dox nanomedicine in HepG2 cells was analyzed by Laser Scanning Confocal Microscopy (LSCM)^19^. The fluorescence signal of Dox from the β-CD-DPPE-Dox nanomedicine was detected by LSCM (TCS SPE, Leica, Germany).

### Animal experiment and blood serum chemistry

#### Animal experiment

The cytotoxicity of β-CD-DPPE micelles *in vivo* and antitumor effects of β-CD-DPPE-Dox nanomedicine were evaluated through tumor-bearing (H22 murine hepatoma cell line) mouse model. When mice tumors grew to 100–200 mm^3^, mice were randomly divided into 3 groups (n = 4 per group) which were PBS (1x) control group, β-CD-DPPE micelles group (3 mg·mL^−1^), β-CD-DPPE-Dox (3 mg·mL^−1^) groups separately. All groups were treated with tail vein injection (3 mg · ml^−1^, 200 μl per time) separately at a set time (0, 2, 4, 6, 8, 10 and 12 days), and PBS (1X, 200 μl) as the control group. At the prescribed time, the tumor size and body weight of mice was measured. After observation for 12 days, the mice were sacrificed, and the tumors were dissected. After dissection, the tumor tissues were soaked in paraformaldehyde for 48 hours, and then the tumor tissues were dehydrated. Then the tumor tissues were paraffin-embedded, sectioned, and finally stained with HE. The tumor tissue sections were observed with the microscope (Olympus CX31).

#### Blood serum chemistry

The experiment mice were injected via tail vein every other day with the 15 mg kg^−1^ sample concentration for 9 days, and the mice blood of every group were collected with the decapitation method on the ninth day. The blood would freeze after 20–30 mins, and then centrifuged for 10 minutes at 4500 r/min, and then the blood serum were collected and placed at 4 °C before use. Blood serum chemistry was tested with auto analyze tester (Siemens Aptio).

The all animal experiment methods were carried out in accordance with the relevant guidelines and regulations of the Laboratory Animal Ethics Committee of Binzhou Medical University. And the all experimental protocols were also approved by the Laboratory Animal Ethics Committee of Binzhou Medical University.

### Statistical analysis

Each set of data in the test was processed with an average value ± the average standard deviation. The significant differences of data were analyzed with the t-test or analysis of variance to determine if there were significant differences in the data. Significant differences were considered when the P value was less than 0.05.

## Results and Discussion

### Synthesis of β-CD-DPPE molecules

Figure S3 was the FT-IR spectrum of β-CD-NH-CO-C_6_H_4_-COOH and β-CD-NH_2_ molecules, and the stretching vibration peak of the carbonyl group (-CO-) in the carboxylic acid group of β-CD-NH-CO-C_6_H_4_-COOH molecules appeared at 1712 cm^−1^, whereas the β-CD-NH_2_ molecule had no peak in this wavenumber, which indicated that the β-CD-NH-CO-C_6_H_4_-COOH molecule was successfully synthesized through the covalently conjugation of CH_3_O-C_6_H_4_-COOH and β-CD-NH_2_ molecules, and successfully hydrolyzed to form the β-CD-NH-CO-C_6_H_4_-COOH molecules. At the same time, NMR spectroscopy data and mass spectrum had also fully proved the β-CD-NH-CO-C_6_H_4_-COOH molecules to be successfully synthesis (See experimental part). Figure S4 was the H-NMR spectra of β-CD-DPPE molecules, the 8.35 ppm peak was the HNMR spectra of -NHCO-C_6_H_6_- group, and the 7.3 ppm peak was the HNMR spectra of β-CD-NH-CO- group, and which proved the β-CD-NH-CO-C_6_H_4_-COOH molecules and the DPPE has been successfully conjugated through the amide reaction, and the 1957.11 peak of MS which was the molecule ion peak spectra of β-CD-DPPE molecules (Fig. S5) also proved the DPPE molecules has been successfully synthesized.

### Characterization of β-CD-DPPE micelles

**Figure 5:**
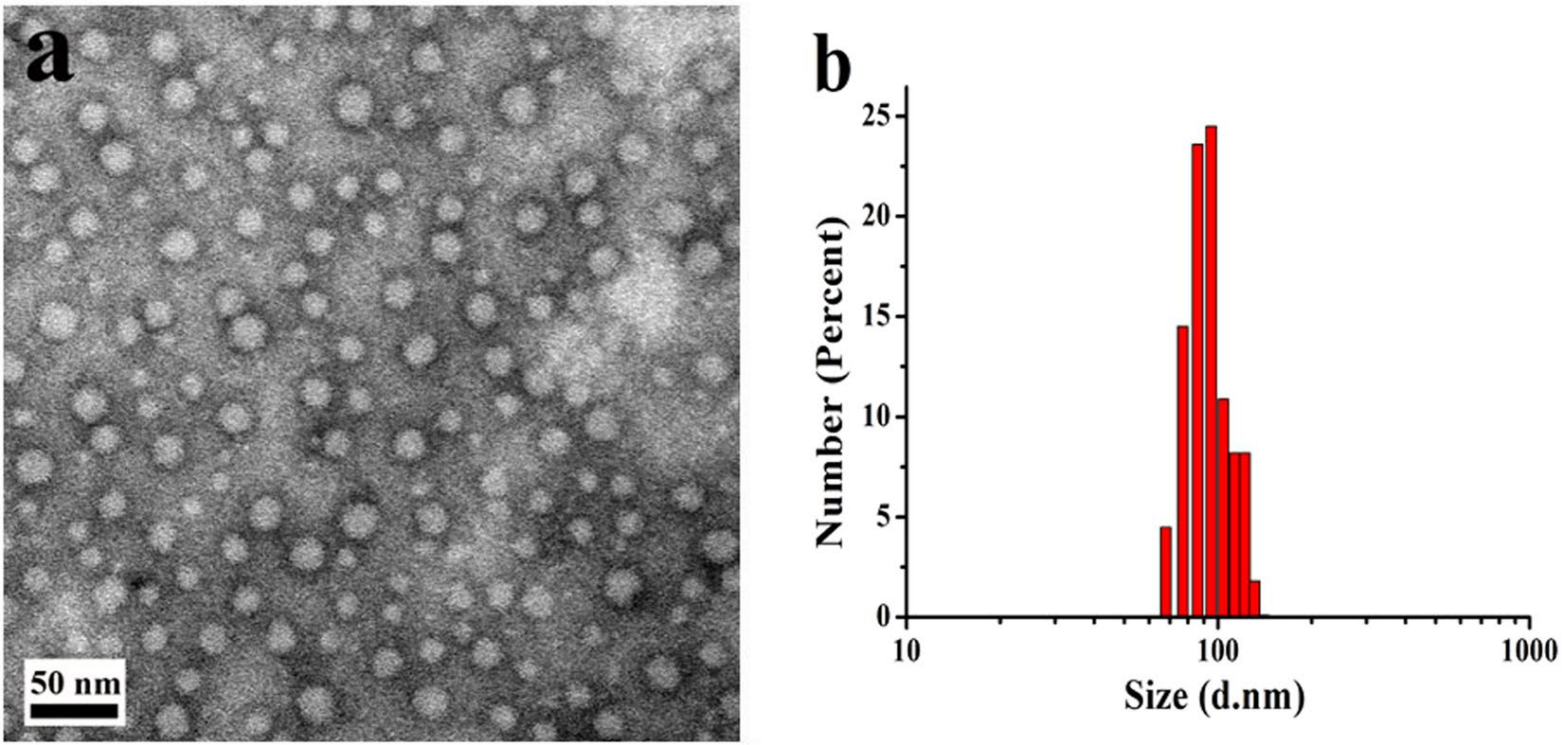
TEM image and size distribution of β-CD-DPPE micelles. (**a**) TEM image of micelles. (**b**) Size distribution of β-CD-DPPE micelles.

β-CD-DPPE molecules are amphiphilic molecules which contain a hydrophilic head group and a hydrophobic long-chain group, and it can self-assemble into micelles in water solution (See the experimental section for preparation of micelles). The CMC value of the micelles exhibits a concentration of 0.01413 mg·mL^−1^ (Fig. S6). TEM pictures showed that β-CD-DPPE could well self-assembled into mono-dispersed, regular spherical micelles with an average particle size about 20 nm (Fig. 5a), and Malvern laser particle size analyzer was used to determine the size and Zeta potential of micelles in aqueous solution, and the results showed that the average particle size of β-CD-DPPE micelles in aqueous solution was 91.0 ± 2.3 nm (Fig. 5b). It was reported in the literature that micelles with a particle size of around 100 nm were more likely to accumulate at tumor sites and had very good EPR effect^20^. The effect is that, compared to free Dox, Dox loaded in this micelles can better aggregate at the tumor site. At the same time, the Zeta potential of the β-CD-DPPE micelles in aqueous solution was −28.53 ± 1.50 mV, which indicated that the micelles has good stability in water, and at the same time, the size change of β-CD-DPPE aggregates was also not obvious in normal saline (10% (V/V) serum) solution with the time increasing (Fig. S7), and which also indicated the micelles has good stability in physiological solution. Generally speaking, the more negative the zeta potential, the more stable it is in water, and it is not easy to aggregate^19^. The proper particle size and negative Zeta potential of β-CD-DPPE micelles favored the aggregation in tumor tissues, and the micelles loaded drug will well interact with cancer cell to better exert its’ anti-cancer and anti-tumor effects.

### Drug entrapment ratio of β-CD-DPPE micelles

Doxorubicin (Dox) is a broad-spectrum antitumor drug, and it can embed in DNA to inhibit DNA replication and transcription, and which is widely used in the treatment of various cancers^21^. However, the damaging effects of Dox on normal tissues have limited its clinical use, such as damaging to kidneys and cardiotoxicity. To solve this problem, Dox can be encapsulated in micelles to avoid inherent side effects. Dox was selected as an anticancer drug model to study the encapsulation ratio and drug release behavior of β-CD-DPPE-Dox nanomedicine *in vitro*. The specific encapsulation process has been described in the experimental section. The Dox was encapsulated by evaporation of organic solvents and rehydration with PBS. The results showed that the encapsulation efficiency of β-CD-DPPE micelles to Dox was 82.3 ± 7.27% and drug loading was 42.86 ± 2.00%. Contrasted with the Dox encapsulation efficiency of β-CD (17.6 ± 7.2%)^20^, β-CD-DPPE micelles has a higher encapsulation efficiency, so we predicted the Dox was encapsulated in both β-CD and micelles.

### Drug release behavior from the β-CD-DPPE-Dox nanomedicine *in vitro*

**Figure 6:**
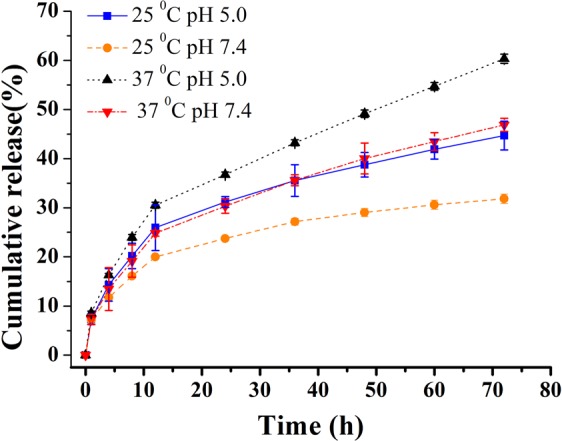
Release behavior of Dox from the β-CD-DPPE-Dox micelles at pH 5.0 and pH 7.4 separately *in vitro* at 25 °C and 37 °C separately.

The release behavior of Dox from β-CD-DPPE-Dox nanomedicine directly affect the effect on cancer cells. The release of Dox from β-CD-DPPE-Dox nanomedicine at different temperatures and different pH conditions were studied as shown in Fig. 6. The release rate of Dox at pH 5.0 was significantly faster than that of Dox at pH 7.4, which indicated that β-CD-DPPE-Dox nanomedicine has a faster release rate in low pH solution. Large amounts of tumor cells require a large amount of energy to proliferation, which results in a large amount of lactic acid produced by anaerobic respiration at the tumor site to reduce the pH of the tumor site. Therefore, β-CD-DPPE-Dox nanomedicine has a faster release rate at lower pH, which can effectively increase the amount of Dox released at the tumor site, and thus it can better act on the tumor tissue. The release rate at 25 °C was significantly slower than that at 37 °C, and there was only a small amount of Dox released at room temperature. From the release curve of Fig. 6, it can be seen that the Dox release is a slow release, and the slow release facilitates the sustained release of Dox to the cancer cells for the purpose of continuous administration.

### Cytotoxicity of β-CD-DPPE micelles and anti-cancer activity of β-CD-DPPE-Dox nanomedicine

**Figure 7:**
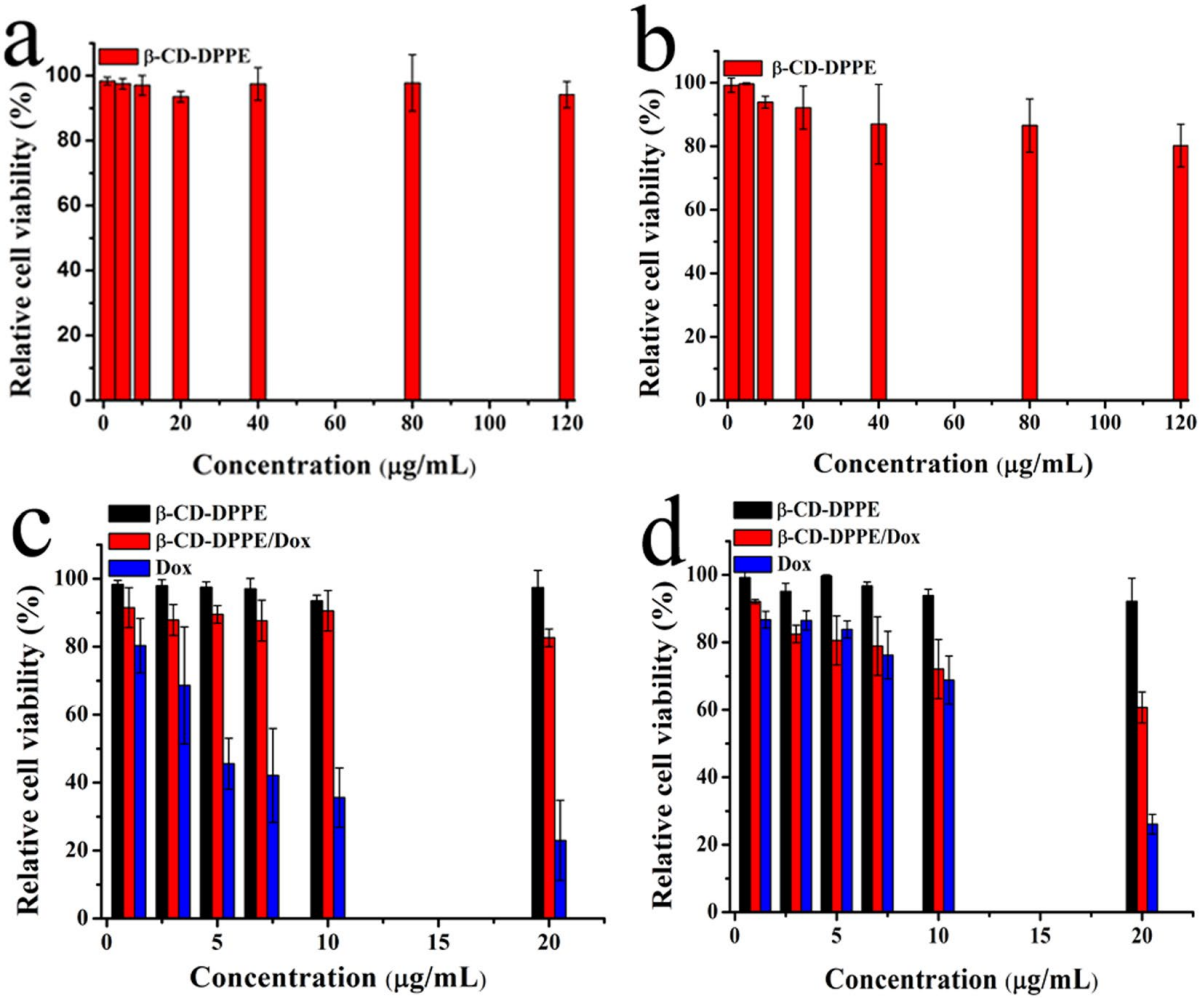
MTT assay after 24 h incubation with HepG2 and MCF-7 cell. (**a**) Cytotoxicity of β-CD-DPPE on HepG2 cell. (**b**) Cytotoxicity of β-CD-DPPE on MCF-7 cell. (**c**) Anti-cancer activity of β-CD-DPPE-Dox liposome nanomedicines on HepG2 cancer cell. (**d**) Anti-cancer activity of β-CD-DPPE-Dox micelles nanomedicines on MCF-7 cancer cell. The significant level was set as P < 0.05.

To assess the toxicological effects of β-CD-DPPE micelles and β-CD-DPPE-Dox nanomedicine, MCF-7 and HepG2 cells were selected as model cells for evaluation by MTT assay. HepG2 cells and MCF-7 cells were cultured with different concentrations of β-CD-DPPE micelles for 24 h separately. The cell viability results were shown in Fig. 7(a,b). It can be seen clearly from the Fig. 7 that the survival rate of both cells was still above 85% when the concentration of β-CD-DPPE micelles was as high as 120 μg·mL^−1^, which indicated that the β-CD-DPPE micelles have low cytotoxicity and good biocompatibility. Figure 7(c,d) were the effect of β-CD-DPPE-Dox nanomedicine on the survival rate of HepG2 and MCF-7 cells respectively. It can be seen that the cytotoxicity of β-CD-DPPE-Dox nanomedicine was significantly lower than that of Dox under the same Dox concentration. The lower cytotoxicity, the better of biocompatibility, and it also reflects the Dox slow release from β-CD-DPPE-Dox nanomedicine. Figure 7(c,d) also showed that the survival rate of HepG2 and MCF-7 cells gradually decreased with the increase of the concentration of β-CD-DPPE-Dox nanomedicine, which suggested that the cell viability of HepG2 and MCF-7 cells were a dose-dependence to β-CD-DPPE-Dox nanomedicine. In general, the cytotoxicity of the drug encapsulated by β-CD-DPPE micelles was reduced, and the biocompatibility was improved, and it had a slow release and sustained drug efficacy.

### Cell uptake

**Figure 8:**
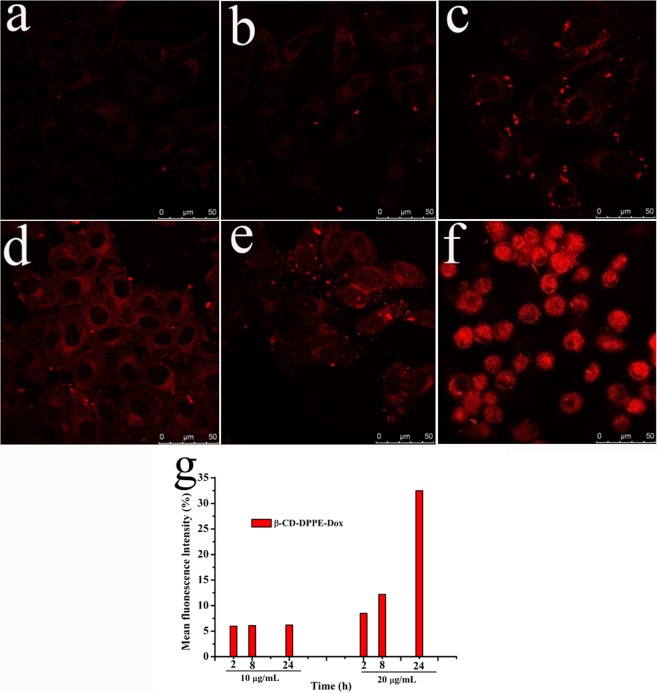
LSCM images of HepG2 cell. (**a**) 10 μg·mL^−1^ β-CD-DPPE-Dox at 2 h; (**b**) 10 μg·mL^−1^ β-CD-DPPE-Dox at 8 h; (**c**) 10 μg·mL^−1^ β-CD-DPPE-Dox at 24 h; (**d**) 20 μg·mL^−1^ β-CD-DPPE-Dox at 2 h; (**e**) 20 μg·mL^−1^ β-CD-DPPE-Dox at 8 h; (**f**) 20 μg·mL^−1^ β-CD-DPPE-Dox at 24 h. (**g**) According to the mean fluorescence intensity (See Fig. S9) acquired the histogram with concentration and time changed.

The distribution of β-CD-DPPE-Dox nanomedicine in HepG2 cells was observed with the LSCM at the concentration 10 μg·mL^−1^ and 20 μg·mL^−1^ separately^22^. The results were shown in Fig. 8. The fluorescence signal for β-CD-DPPE-Dox nanomedicine with 10 μg·mL^−1^ were observed at 2 h, 8 h, and 24 h respectively. It was different from the Dox group (Fig. S8) which could be rapidly uptake by HepG2 cells, and the fluorescence signal of β-CD-DPPE-Dox nanomedicine group was gradually enhanced with the increasing of time, and the fluorescence signal of the Dox from the β-CD-DPPE-Dox nanomedicine firstly appeared on the surface of the HepG2 cell membrane as shown at 2 h (Fig. 8a), and then entered the cytoplasm as shown at 8 h (Fig. 8b), and finally entered the nucleus with very small amount of Dox as shown at 24 h (Fig. 8c), however there were not significant effect on the cell morphology. When the concentration of β-CD-DPPE-Dox was 20 μg·mL^−1^, it can be seen that the fluorescence signal at 2 h (Fig. 8d), 8 h (Fig. 8e), and 24 h (Fig. 8f) was significantly higher than that at a concentration of 10 μg·mL^−1^. The intracellular Dox fluorescence signal gradually increased with the time increasing, and finally Dox fluorescent signal obviously appeared in the nucleus as shown at 24 h, and the cell morphology had turned round, crinkle, and appearing the apoptotic state. In conclusion, with the increase of the concentration of β-CD-DPPE-Dox nanomedicine and the prolongation of time, Dox of β-CD-DPPE-Dox nanomedicine can be well released into the cytoplasm, even into the nucleus and induce the cancer cell apoptosis.

The accurately quantification for the accumulation of Dox from the β-CD-DPPE-Dox nanomedicine in cell was analyzed with the FC to evaluate the uptake^23^. It was explored that different doses of β-CD-DPPE-Dox nanoparticles enter HepG2 cells at different times as shown in Figs 8g and S9. The fluorescence intensity at both concentrations (10 μg·mL^−1^ and 20 μg·mL^−1^) increased with the prolongation of time, the bigger of the concentration, the stronger the fluorescence intensity (Figs 8g and S9). It indicated that the uptake to β-CD-DPPE-Dox nanomedicine by HepG2 cells was dose-dependent and time-dependent, the conclusion was consistent with the result of LSCM.

### Anti-tumor studies *in vivo*

**Figure 9:**
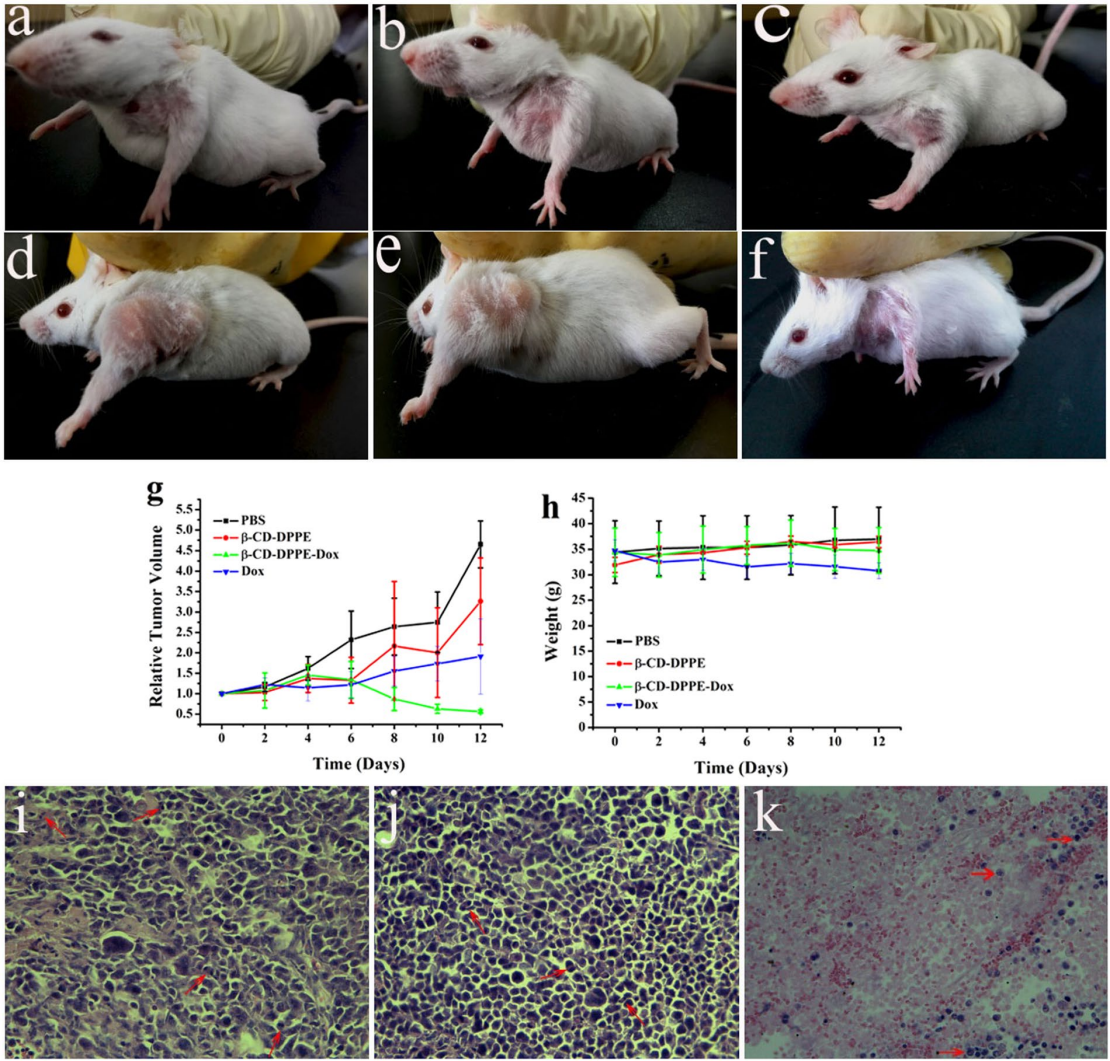
The therapeutic effect of β-CD-DPPE-Dox micelles nanomedicine *in vivo*. (**a**) Tumor before injected the PBS. (**b**) Tumor before injected the β-CD-DPPE micelles. (**c**) Tumor before injected the β-CD-DPPE-Dox micelles nanomedicine. (**d**) Tumor injected the PBS after 12 days. (**e**) Tumor injected the β-CD-DPPE micelles after 12 days; (**f**) Tumor injected the β-CD-DPPE-Dox micelles after 12 days. (**g**) The tumor volume change with the time increase. (**h**) The weight change with the time increase. **(i**) Tissue section of tumor was injected PBS after 12 days. (**j**) Tissue section of tumor was injected β-CD-DPPE micelles after 12 days. (**k**) Tissue section of tumor was injected β-CD-DPPE-Dox micelles nanomedicine after 12 days.

In order to study the therapeutic effect of β-CD-DPPE-Dox nanomedicine *in vivo*, tumor-bearing mice (Mice injected with H22 cancer cells) were used as a research model^24^. To PBS group (Fig. 9a,d), it can be seen that the tumor volume became significantly larger and spread from the forelimbs to the entire forelimb, and even extend to the back after 12 days of injection; Fig. 9b,e show that the tumor volume was also significantly larger, and the tumor also spreads to the back after 12 days of β-CD-DPPE micelles injection; however, the tumor volume of the β-CD-DPPE-Dox nanomedicine group and the Dox group (Fig. S10) was significantly reduced after 12 days of injection (Fig. 9c,f), and tumor proliferation was not observed, which suggested that β-CD-DPPE-Dox nanomedicine can release Dox and act on the tumor to achieve the effect of inhibiting tumor growth and proliferation *in vivo*. The detailed changes in the relative volume of tumors are shown in Fig. 9g. It can be intuitively seen that β-CD-DPPE micelles have no inhibitory effect on tumors as compared with the control group. The tumor volume of β-CD-DPPE-Dox nanomedicine group began to decrease after 6 days of administration which was similar to the Dox group (Fig. S10). With the prolongation of time, the effect of β-CD-DPPE-Dox nanomedicine on tumor growth was more prominent, and the tumor volume became smaller and smaller. What is even more gratifying is that there was no significant change in body weight of mice after injection of β-CD-DPPE-Dox nanomedicine (Fig. 9h), which indicated that β-CD-DPPE micelles and β-CD-DPPE-Dox nanomedicine have good biocompatibility, low toxicity, and good therapeutic effect on tumors. The details changes in the tumor tissues were analyzed by histopathology. Figures 9(i–k) and S10 are the microscope observation pictures after H&E staining to tumor tissue. The changes of the tumor histological sections of PBS group (Fig. 9i) and the β-CD-DPPE micelles group (Fig. 9j) is similar. The tumor cells of the PBS group showed good morphology, and cell proliferation and division was vigorously, and we can see that the cells were in the proliferative division stage (Indicated with the red arrow); The β-CD-DPPE micelles group showed also very good cell morphology, and the cell density was greater than that of the PBS group, which indicated that the cancer cells growth was better, and the proliferation and division of cells were more prosperous (Fig. 9j, red arrow), and it may be the role of β-CD and DPPE units in β-CD-DPPE micelles. Because β-CD has partial sugar properties, and the DPPE has the property of phospholipids, so cancer cells uptake extra energy relative to the growth of cancer cells, resulting in even more robust growth and proliferation of cancer cells. The tumor tissue and tumor cancer cell changes of β-CD-DPPE-Dox nanomedicine group are shown in Fig. 9k. It can be clearly seen that the cancer cell appeared shrinks, rounds, nucleus pyknosis simultaneously which is similar to Dox group (Fig. S10). The reason may be Dox in β-CD-DPPE-Dox nanomedicine could be released and induce the cancer cells apoptosis very well, resulting in extensive necrosis of tumor tissue, and even cancerous tissues without cancerous structures appeared. The above results fully demonstrate that β-CD-DPPE-Dox nanomedicine can significantly inhibit the growth of tumor without affecting the body weight of mice, and effectively induce apoptosis of cancer cells in the tumor tissue, and lead to large area of necrosis of tumor tissue, and eventually make the tumor volume significantly reduced to achieve an effective treatment of tumors.

Liver function (Table S1) test showed that ALT (Alanine aminotransferase), ALB (Albumin) and TBIL (Total bilirubin) in NS (Normal Saline) group were higher than those in other groups, which indicated that the tumors in NS group caused severe liver damage in mice, especially in ALT group. The small change of GLD in all groups indicated that the tumor had no significant effect on the immune system of mice. At the same time, it could be seen from the above data that the β-CD-DPPE-Dox which was formed through the load of β-CD-DPPE micelles to Dox greatly reduced the damage of Dox to the liver and played a important role in protecting the liver. The renal function (Table S2) of NS group was severely damaged by tumors, and the contents of urea nitrogen (BUN) and uric acid (UA) in Dox group were still high due to the side effects of adriamycin. The contents of BUN and UA in β-CD-DPPE group and β-CD-DPPE-Dox group were lower than those in NS group, which indicated that the formation of β-CD-DPPE-Dox reduced the damage of Dox to the kidney and β-CD-DPPE micelles played an anti-cancer role while protecting the kidney. It could be seen from the myocardial enzyme tests (Table S3) that Dox had serious cardiotoxicity, and NS group has higher AST and α-HBDH content, which results in the occurrence of myocardial infarction due to the compression of the heart by tumors. Compared with NS group and Dox group, the contents of AST and α-HBDH of the β-CD-DPPE-Dox group were significantly lower than those in the NS saline group and the Dox group, which indicated that the β-CD-DPPE-Dox nanomedicine reduced the cardiotoxicity of Dox and had antintumor effect.

## Conclusion

Herein, a novel amphiphilic β-CD-DPPE molecule was successfully synthesized by β-CD modification to the phospholipid molecule DPPE, and it can self-assembly into a spherical β-CD-DPPE micelles with uniform size 91.0 ± 2.3 nm in aqueous solution, and the hydrophilic shell of β-CD increase the water solubility of DPPE, and the hydrophobic cavities which could load the fat-soluble drugs to the bioavailability of loaded drugs and reduce the drug cytotoxicity. The size of β-CD-DPPE aggregates is consistent as the basic requirements of a drug delivery system in body. The micelles has a higher Dox loading rate which is 82.3 ± 7.27%, and the Dox release behavior from β-CD-DPPE-Dox nanomedicine is temperature, pH responsiveness and a slow release profile which prolong the drug circle time in body and increase the drug bioavailability. Cell experiments showed that the toxicity of β-CD-DPPE micelles was extremely low, and the uptake of β-CD-DPPE-Dox nanomedicine by cancer cells was time and dose dependent, and at the same time the β-CD-DPPE-Dox nanomedicine could effectively induce cancer cell apoptosis. Animal experiments showed that β-CD-DPPE-Dox nanomedicine could effectively induce cancer cell apoptosis in tumor tissue and further result in necrosis of tumor tissue without affecting mouse body weight, which make the tumor volume become smaller, even vanish, and it further proved that β-CD-DPPE-Dox nanomedicine could effectively prevent tumor growth, and achieve the treatment purpose of tumors. Blood serum chemistry showed that the β-CD-DPPE-Dox nanomedicine could obviously reduce the toxicity of Dox to liver, renal and myocardial enzyme, which indicated that the β-CD-DPPE micelles was very ideal drug delivery system. It is hoped that this novel micelles will play a constructive role in reducing the toxic side effects of antitumor drugs and increasing the water solubility and biocompatibility of drugs.

## Supplementary information

Supporting Information
