# Supplementary material for: Preparation of β-CD-DPPE-Dox Nanomedicine and Its’ Application as the Anticancer and Antitumor Drug
Source: Sci Rep. 2019 Sep 20;9:13670. doi: 10.1038/s41598-019-50162-8 (PMC6754386; doi:10.1038/s41598-019-50162-8)
Supplement: Supplementary file 1 — Supporting Information [file 41598_2019_50162_MOESM1_ESM.docx]

**Supporting Information**

**Preparation of β-CD-DPPE-Dox Nanomedicine and Its' Application as the Anticancer and Antitumor Drug**

Miaomiao Yan^†^, Anran Cai^†^, Jing Li^†^, Meixiu Xin, Mingying Liu, Chunhua Wang^*^ and Guangcheng Wei^*^

Department of Pharmacy Science, Binzhou Medical University, Yantai, 264003, China.

† These authors are equal to the contribution of this work.

E-mail: weiguangcheng2004@126.com; chhuawang@126.com


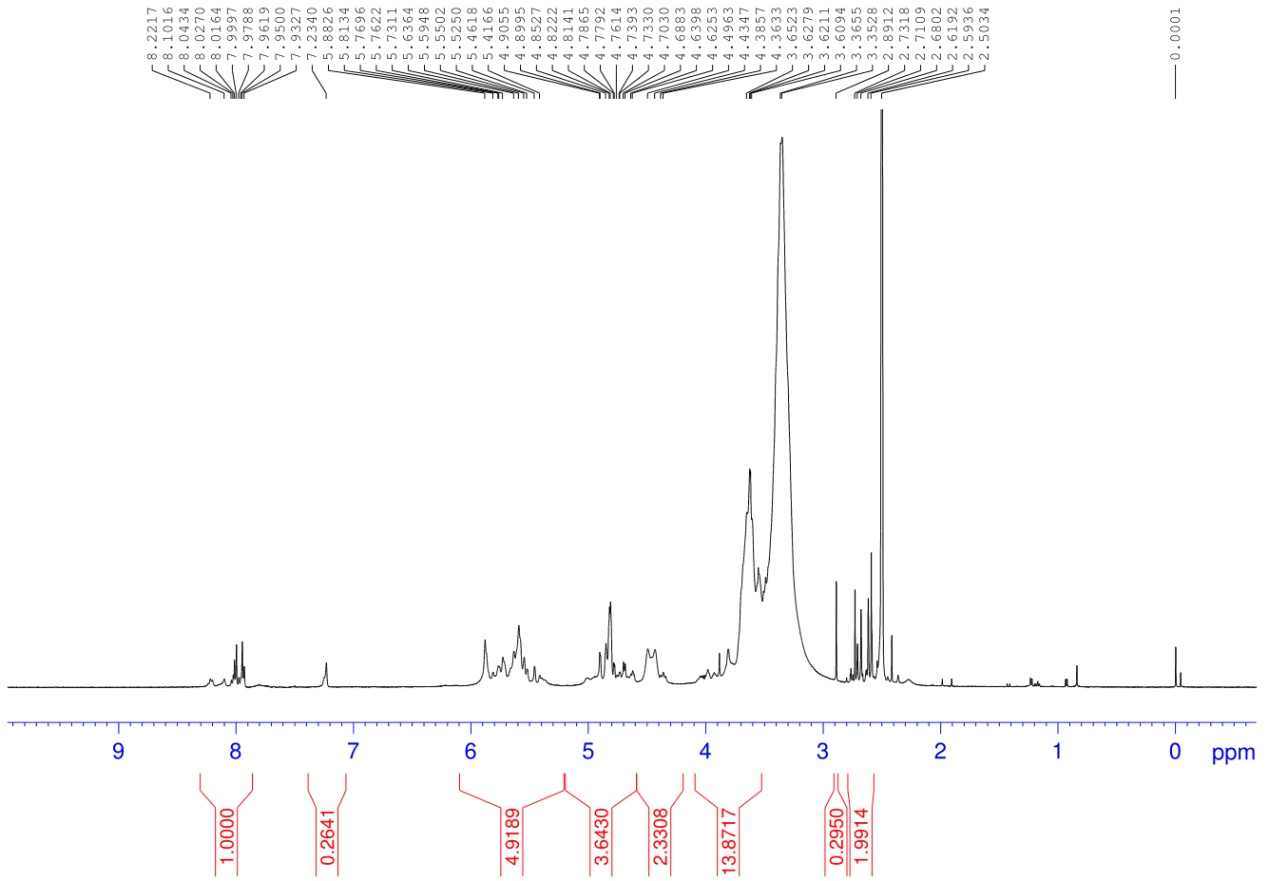


**Figure S1.** The ^1^HNMR spectrum of β-CD-NH-CO-C_6_H_4_-COOH molecule.

**Figure S2.** The mass spectrum of β-CD-NH-CO-C_6_H_4_-COOH molecule.

**Figure S3.** The FT-IR spectrum of β-CD-NH-CO-C_6_H_4_-COOH molecule.


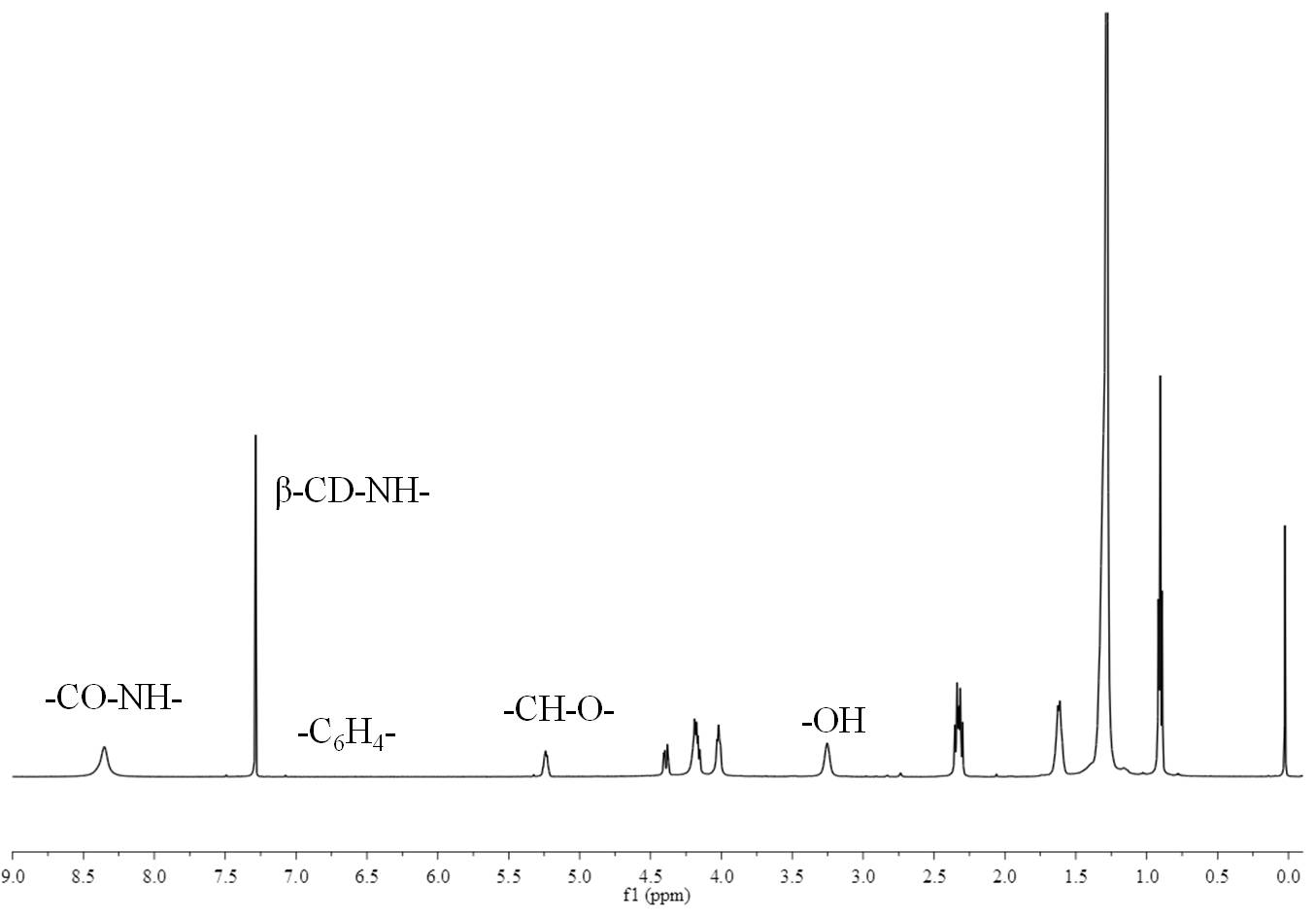


**Figure S4.** The ^1^HNMR spectrum of β-CD­­-DPPE molecule.

**Figure S5.** The MS spectrum of β-CD­­-DPPE molecule.

**Figure S6.**  CMC determination of β-CD-DPPE molecules in aqueous solution

**Figure S7**. The size change of β-CD-DPPE aggregates in normal saline（10%（V/V）serum）solution.


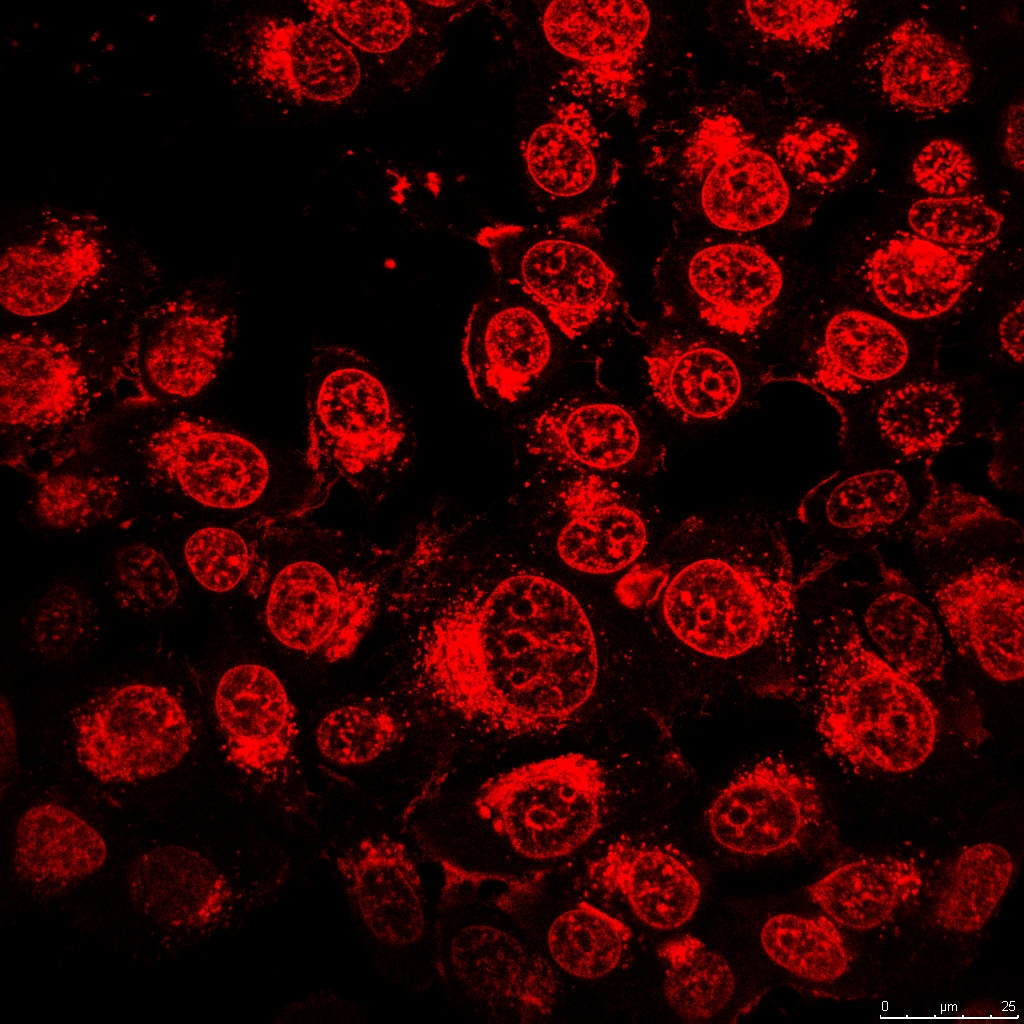


**Figure S8.** 10μg·mL^-1^ Dox group LSCM images of HepG2 cell at 2h.


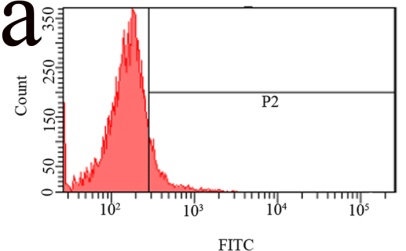

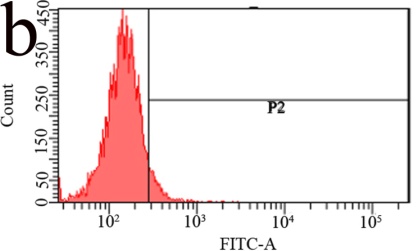

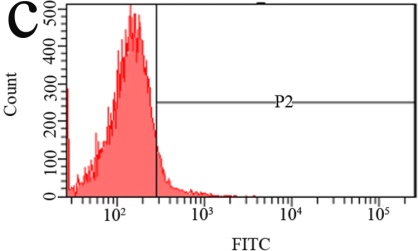


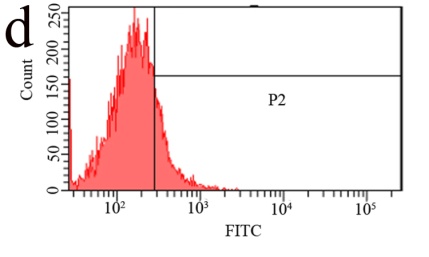

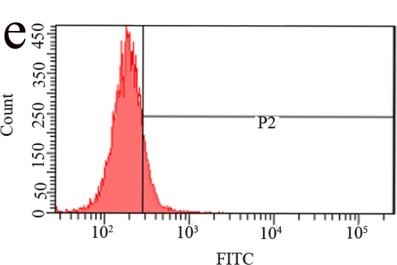

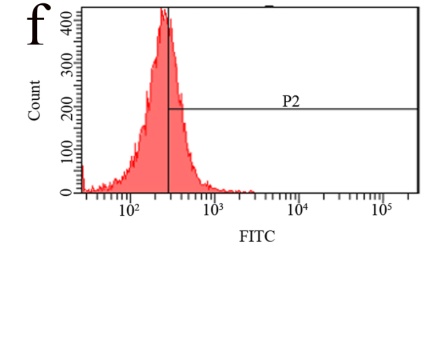


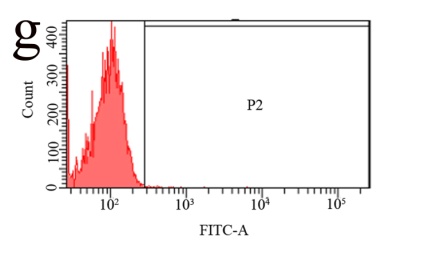


**Figure S9.** Cell uptake assay was analyzed with the flow cytrometry. (a) 10μg·mL^-1^ β-CD-DPPE-Dox micelles was incubated with HepG2 cell for 2h; (b) 10μg·mL^-1^ β-CD-DPPE-Dox micelles was incubated with HepG2 cell for 8h;(c) 10μg·mL^-1^ β-CD-DPPE-Dox micelles was incubated with HepG2 cell for 24h; (d) 20μg·mL^-1^ β-CD-DPPE-Dox micelles was incubated with HepG2 cell for 2h; (e) 20μg·mL^-1^ β-CD-DPPE-Dox micelles was incubated with HepG2 cell for 8h; (f) 20μg·mL^-1^ β-CD-DPPE-Dox micelles was incubated with HepG2 cell for 24h; (g) Control group


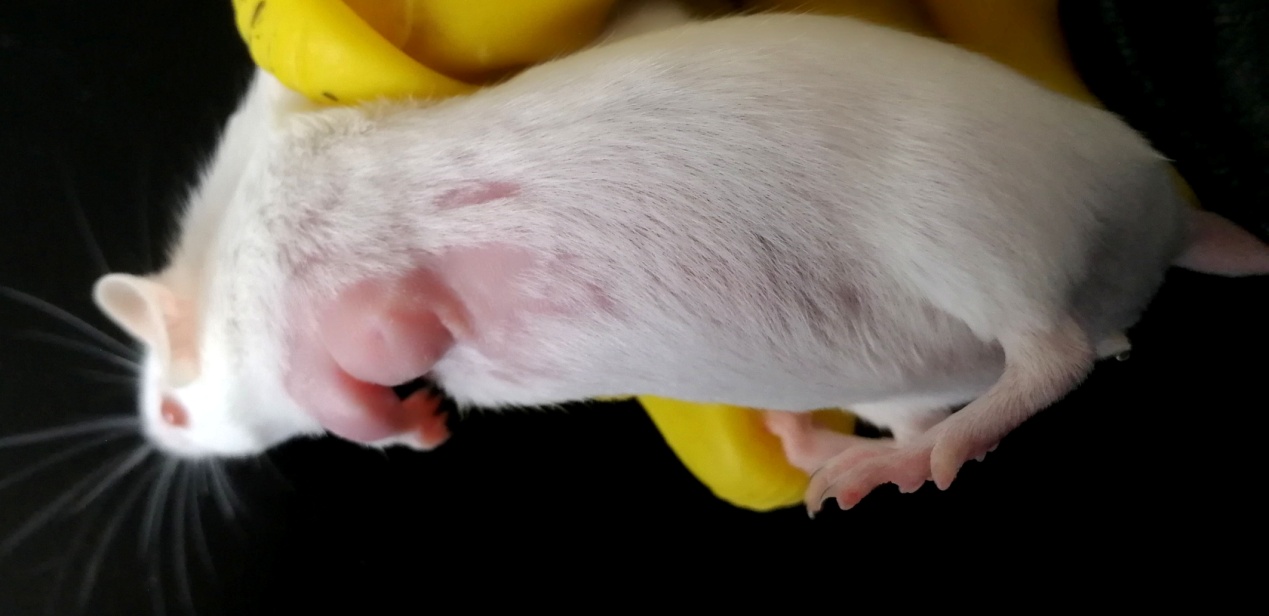


(a)


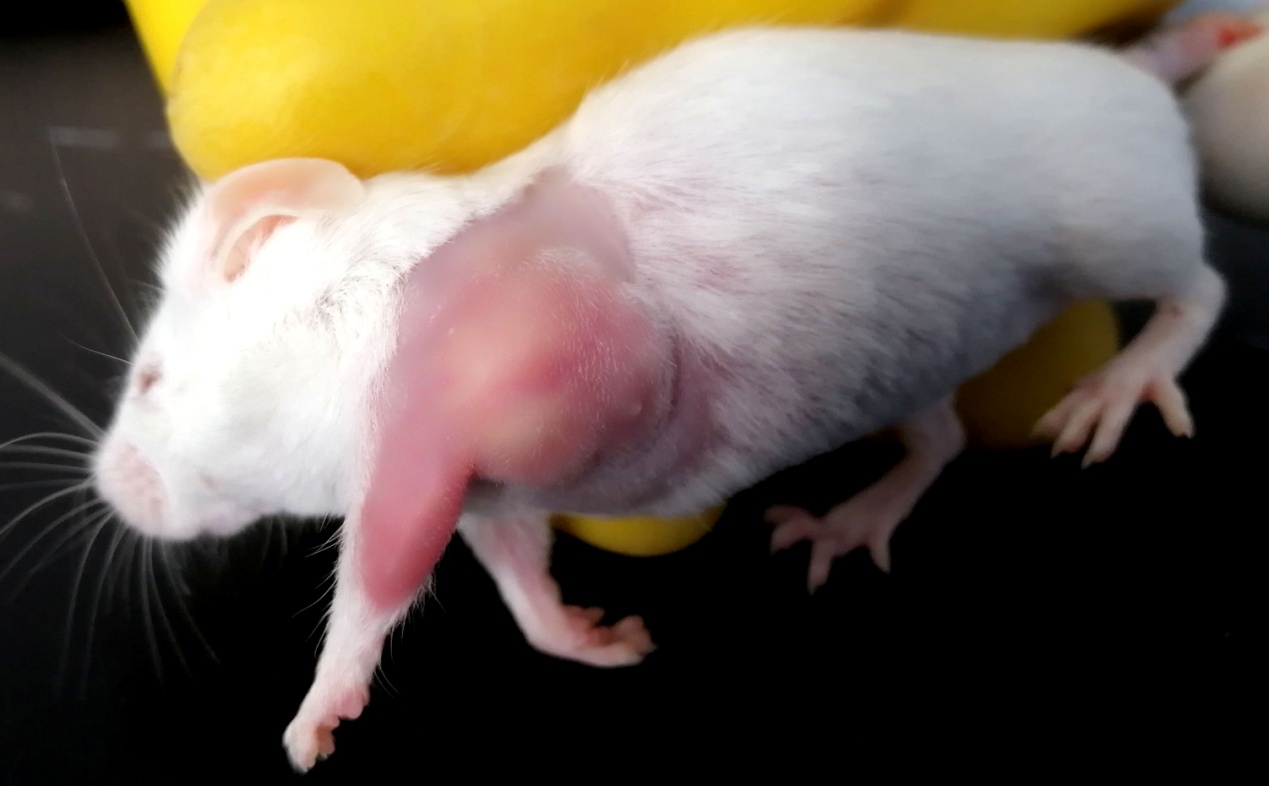


(b)


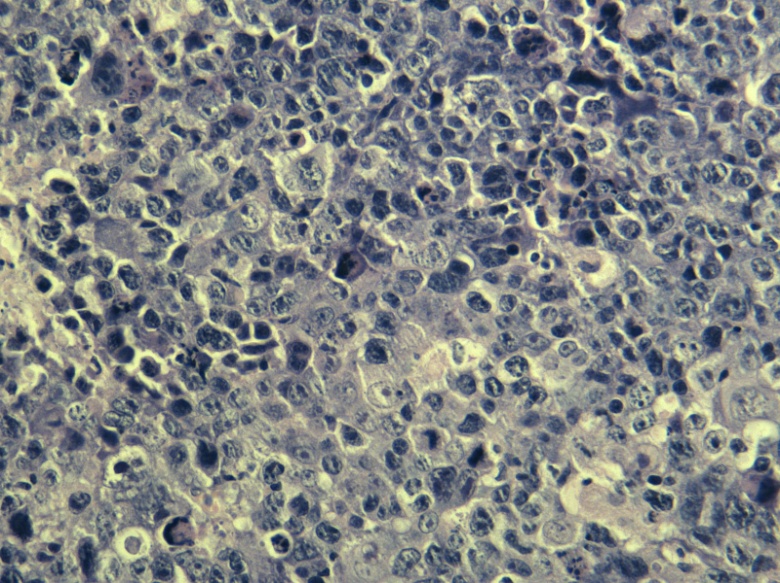


(c)

**Figure S10**. The therapeutic effect of Dox in vivo. (a) Tumor before injected the Dox; (b) Tumor injected the Dox after 12 days; (c) Tissue section of tumor was injected Dox micelles after 12 days

**Table S1.**  Liver function tests.

| **Liver function tests** | **NS**  **group** | **Dox**  **group** | **β-CD-DPPE**  **group** | **β-CD-DPPE-Dox**  **group** |
| --- | --- | --- | --- | --- |
| ALT(U/L) | 398.1±22.98 | 130.8±25.45 | 241.1±22.45 | 104.6±24.41 |
| ALB(g/L) | 22.3±2.87 | 13.6±2.67 | 21.2±2.70 | 21.3±2.94 |
| GLD(g/L) | 38.6±3.65 | 39.1±2.82 | 38.3±2.33 | 41.2±3.12 |
| TBIL(μmol/L ) | 5.0±0.21 | 4.1±0.33 | 4.7±0.16 | 4.4±0.11 |

ALT: Alanine aminotransferase; ALB: Albumin; GLD : Globulin; TBIL: Total bilirubin

**Table S2.** Renal function tests.

| **Renal function**  **tests** | **NS**  **group** | **Dox**  **group** | **β-CD-DPPE**  **group** | **β-CD-DPPE-Dox**  **group** |
| --- | --- | --- | --- | --- |
| BUN(mmol/L) | 8.06±0.28 | 7.54±0.34 | 6.99±0.17 | 7.23±0.21 |
| UA(μmol/L ) | 269.1±21.39 | 261.5±24.33 | 176±21.01 | 229.1±20.18 |

BUN: Urea nitrogen; UA: Uric acid

**Table S3.** Myocardial enzyme tests

| **Myocardial enzyme tests** | **NS**  **group** | **Dox**  **group** | **β-CD-DPPE**  **group** | **β-CD-DPPE-Dox**  **group** |
| --- | --- | --- | --- | --- |
| AST(U/L) | 789.9±45.67 | 1997.9±150.64 | 579.7±56.87 | 510.1±38.47 |
| α-HBDH(U/L) | 2181.9±180.14 | 2207.68±150.61 | 1791.3±145.27 | 1896.7±165.34 |

AST : Aspartate aminotransferase; α-HBDH: α-Hydroxybutyrate Dehydrogenase
